# End Stage Renal Disease: Seroprevalence of Hepatitises B and C along with Associated Aetiology and Risk Factors in Children

**DOI:** 10.1155/2015/936094

**Published:** 2015-08-05

**Authors:** Syed Raza Shah, Muhammad Shahzeb Khan, Muhammad Tanveer Alam, Adnan Salim, Mehwish Hussain, Areeba Altaf

**Affiliations:** Dow University of Health Sciences (DUHS), Baba e Urdu Road, Karachi 75500, Pakistan

## Abstract

*Background*. End Stage Renal Disease (ESRD) normally requires dialysis or transplantation for survival. Since ESRD patients are on long term dialysis, infections such as Hepatitis B (HBV) and Hepatitis C (HCV) are commonly reported. *Methods*. This was a retrospective study carried out at a government hospital during a 12-month period from January 2013 to December 2013. The data was collected using a predesigned pro forma to note the etiology, gender, age, and HBsAg and anti-HCV test result of each patient. *Results*. 444 children suffering from ESRD were included in our analysis. The mean age of sample was 12.7 ± 4.1 years. Sixty percent (*n* = 262) of the children were boys. The most common etiology of ESRD was kidney stones (*n* = 44, 29.3%). HBV was positive in 11 children (2.5%) while HCV was positive in 13 (2.9%). *Conclusion*. This study asserts the need for carrying out further work to confirm these findings and expand our recommendations. It is imperative to reliably determine the burden of HBV and HCV disease and to determine the aetiology of their spread especially in children with ESRD.

## 1. Introduction

End Stage Renal Disease (ESRD), the stage 5 of chronic kidney disease, normally requires dialysis or transplantation for survival. The burden of ESRD in children is lesser as compared with adults; however, if ESRD occurs in children, consequences can be catastrophic. It should be pointed out that children on dialysis have 30 to 150 times higher mortality rates compared with the general children population [1]. Children with ESRD end up dying from variety of causes such as cardiovascular diseases, life threatening infections, and malignancy [2].

Since ESRD patients are on long term dialysis, infections such as Hepatitis B (HBV) and Hepatitis C (HCV) are commonly reported. HBV is a major cause of mortality in such patients [3]. Although vaccination has significantly reduced mortality rates, dialysis can shorten the duration of immunity against HBV by lowering the protective antibody levels in children who were vaccinated as infants [3, 4]. Moreover, the duration of dialysis has high predictive risk for HCV infections. This was consistent with a study in which all patients who were anti-HCV positive had been on dialysis for a mean of 105 months [5]. Furthermore, a study also reported a steady rise of the HCV antibody titres in children during the period of dialysis treatment [6]. This may be due to the number of blood transfusions at the time of haemodialysis which has been shown to be a significant risk of HCV infection [7].

Many studies have been conducted to determine the rate of HBV and HCV infections among the paediatric ESRD population. For instance a study conducted in Saudi Arabia showed that the prevalence of anti-HCV in children with ESRD was 45% compared with the prevalence of 1% among the controls [3]. Similarly, another study showed that the prevalence (11.2%) of anti-HCV was much higher in children with chronic renal disease. A study conducted back in 1985 on patients aged between 2 and 18 years with chronic renal failure showed a seroprevalence of 66.7% for HBsAg [8].

Not much data is available on this subject from our part of the world due to nonexistence of a centralized registry. While the incidence of ESRD in the United States is declining (a 5.8% decrease in 2012), it continues to be rising in Pakistan, with an estimated annual incidence of >100 new cases per million population [9, 10]. Epidemiological data on HBV and HCV infection is therefore important for strategies to tackle the spread of the disease. Moreover, it is imperative to reliably determine the burden of HBV and HCV disease, to determine the etiology of spread especially in children with ESRD, to identify any areas with higher endemicity than the rest of the country and to understand the risk factors associated with its transmission. The primary objective of our study was to determine the frequency rate of HBsAg and anti-HCV among children with ESRD while the secondary objective was to determine the etiology, gender, and age groups that are predominantly infected.

## 2. Methods

This was a retrospective study carried out at a government hospital during a 12-month period from January 2013 to December 2013. All subjects' information was kept confidential and a written informed consent was obtained from each participant. All ethical responsibilities were met in accordance with Helmshki law. Since our government hospital is located in the center of the city where people come from all over the city, a good representative sample of the entire city was collected and analyzed.

The sample population included all those children under the age of 18 years who were documented cases of ESRD. ESRD was defined as individuals with a glomerular filtration rate (GFR) of <15 mL/min/1.73 m^2^ or with signs and symptoms of kidney failure. The latter included patients who were currently on dialysis or patients who underwent transplantation.

Blood samples were collected. HBsAg and anti-HCV were tested by in vitro immunochromatographic one step assay designed for qualitative determination. The data was collected using a predesigned pro forma to note the aetiology, gender, age, and HBsAg and anti-HCV test result of each subject. Data was collected by three investigators who were also coauthors of the study. After explaining the purpose of the study, each participant was asked to fill the consent form. Two independent authors queried the data from the database to ensure no error was made. The results were cross verified among the 2 authors to ensure integrity of data. Any discrepancy was solved through mutual consensus.

### 2.1. Statistical Analysis

IBM SPSS v. 21 (SPSS Inc., Illinois, US) was used to analyze the data. Mean ± standard deviation was computed to describe continuous variable. Age was classified into 3 categories, namely, 1–9 years, 10–14 years, and 15–18 years. Frequencies and percentages were calculated for presenting categorical variables such as demographic profile and clinical status of patients. Age based stratified analysis was also performed to observe pattern of clinical profile among ESRD children.

## 3. Results

**Figure 1:**
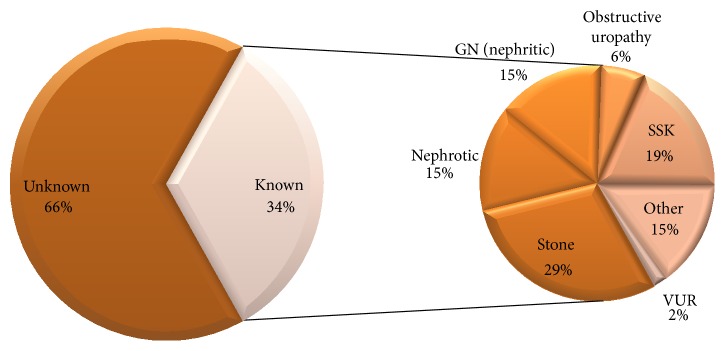
Percentage distribution of Aetiology of ESRD among children.

Our sample population consisted of 444 children suffering from ESRD. The mean age of children was 12.7 ± 4.1 years. 20% (*n* = 91) of the children were less than 10 years of age while 39% (*n* = 174) were in the age group 15–18 (Table 1). 60% (*n* = 262) of the children were boys. The reason for ESRD was known for 33.8% (*n* = 150) patients only (Figure 1). The most common known aetiology of ESRD was kidney stones (*n* = 44, 29.3%), followed by small shrunken kidneys (*n* = 28, 19%) (Table 2).

HBV was positive in 11 children (2.5%) while HCV was positive in 13 (2.9%) (Table 2). The proportion of HBV positivity was found to be 1.1% (*n* = 1) among children less than 10 years. This proportion was higher in children with the age groups 10–14 and 15–18 years with 2.8% (*n* = 5) and 2.9% (*n* = 5), respectively. On the other hand, HCV was positive in 2.2% (*n* = 2), 1.7% (*n* = 3), and 4.6% (*n* = 8) of the children aged 1–9 years, 10–14 years, and 15–18 years, respectively (Table 3).

Table 3 shows age based stratified analysis for secondary outcome variables such as aetiology. The aetiology varied among different age groups. Kidney stones were the most common cause for ESRD among all stratified age groups. Children under the age of 10 years had the highest percentage of kidney stone being the reason for their kidney failure (*n* = 10, 11%) followed closely by the 15–18 years age group (*n* = 18, 10.3%).

## 4. Discussion

The prevalence of HBV and HCV in our sample population is 2.5% and 2.9%, respectively. A major hurdle in recording the prevalence of HBV and HCV in the Pakistani population is the lack of reporting to a national database. A study showed that the prevalence of HCV among the general population is 4.5% [11], while another study estimated that 17 million people in Pakistan are affected [12] compared with the global reported prevalence of less than 3%, 1.8% in Europe, and 2.3% in USA [13]. Prevalence of HBV has been shown to be 2.5% in the general population [14]. A few studies carried out in the paediatric population by Khan [15], Parker et al. [16], and Hyder et al. [17] showed that the rates of HCV are 4.09%, 1.3%, and 0.58%, respectively, while a newer study determined the prevalence to be 3.3% in the age group 9–19 years [18], while a review showed the incidence of HBV as 1.9–3.6% [19].

Keeping the route of infection in mind, some reasons for the higher prevalence of these infections in our country can be deduced. Majority of our population obtains their haircut and facial shaves from unhygienic barber shops, where the practice of using fresh blades for each individual is dubious at best. While this dangerous act used to be much more common, awareness regarding the hazards has reduced it to a great extent. There still remains a lot of work to be done, as these practices have not been completely abolished and many people do not recognize the significance of needle-stick injuries, proper disposal of used sharp objects, sharing of toothbrushes, and unsafe blood donations, apart from the pervasive presence of intravenous drug abuse. It is possible that many of the children are infected either directly through these injuries or accidents or through vertical transmission from their mothers.

Hepatitis infections in ESRD patients, especially children, represent a special subset of the population. The inflicted individuals have to undergo maintenance peritoneal dialysis, haemodialysis, or renal transplantation. All these are associated with risks of blood-borne infections being transmitted, among other complications. Being on dialysis means repetitive exposure to potentially infected instruments and hence increased risk of infections. Studies from different regions show varying trends of HBV and HCV infection in these patients. A Senegalese study showed 5.6% prevalence of HCV in ESRD patients [20], while in Libya it was shown to be 31.1%, in Germany 6.1%, in Saudi Arabia 50%, and in Turkey 20.2% [21]. On the other hand, the observed prevalence of HBV infection in ESRD patients was 2.6% in Libya, 4.1% in Europe, 2.2% in Japan, and 2.4% in USA [21]. A local study revealed frequency of HBV as 2.1% and HCV as 16.4% [22]. While these studies mostly state the frequencies among the adult populations suffering from ESRD, some researchers have worked on the paediatric ESRD patients too. From these, the prevalence in Saudi population is 45% for HCV and 15% for HBV [3], in Italian population 15% for HCV [7], and in the Egyptian paediatric ESRD population 5.9% for HBV and 94.1% for HCV [23]. All these report a much higher incidence of the investigated diseases as compared to our study. As the risk of being infected by blood-borne viruses increases with exposure, patients who are on dialysis for longer periods are at higher risk than those being started on dialysis recently. This would lead to the expectation that children with ESRD would be less likely to be infected as compared to adults with the same affliction since they have been enduring dialysis longer.

We further found that the aetiology of ESRD in our population was unknown in 66.2%. Among the known causes, the most common in our sample was urolithiasis. The main causes of ESRD in children are quite different from those in adults. Diabetes and hypertension being common culprits in the latter [24]. As far as the aetiology in children is concerned, there is a marked variety between countries and regions. While congenital anomalies and hereditary nephropathies are reported from developed nations, infections and other acquired causes make up the majority in developing countries [25, 26]. The paediatric aetiology in our population remains mostly unknown, with a high incidence of urinary tract stones which is in contrast to that reported in Western countries. Stone formation leading to renal failure has a high incidence in our region [27]. A study conducted in Karachi shows nephrolithiasis as the major factor in 16%, glomerulonephritis in 26%, and unknown in 50% [28] compared with hereditary/congenital disorders (35.9%) and glomerular disease (21.5%) in USA [9]. This is consistent with our findings which show stone formation as the leading known cause of ESRD in children.

Pakistan, among other countries of the Afro-Asian belt, has one of the highest incidences of urolithiasis in the world. This entity is observed both in adults and in children and is often neglected which leads to patients presenting with late disease; a challenge to treat [15]. Paediatric urolithiasis in Western countries occurs mainly on the backdrop of metabolic abnormalities, anatomical anomalies, and infections while the preponderant etiological basis in developing nations is not clearly defined [29]. It has been shown that there is a genetic component involved in increasing the risk of stone formation, and dietary and environmental components also contribute to the metabolic abnormalities leading to this phenomenon. Relation of physical activity and fluid intake too is well documented [30]. The importance of urolithiasis as a cause of ESRD is based on the fact that it is quite easily preventable and manageable, hence reducing the burden of patients developing renal failure.

Extrapolating on these evidences, one can make some intelligent guesses regarding the role of environment and diet on our results. In a subtropical region with the sun shining almost the whole year, high temperatures, and humidity, it is no surprise that people living in Pakistan, especially the southern coastal areas, arid Balochistan, and lower Punjab, tend to be dehydrated. Increased consumption of salt is common in our society and, with a significant portion of the population not able to afford meat, consumption of vegetables with the resultant load of oxalate provides additional risk factors. An increase in dietary calcium, associated with dairy consumption, reduces the formation of oxalate stones, which could be a possible contributory factor in those with poor feeding habits [31]. These points can help formulating advice to high-risk patients and the population in general regarding the potential prevention of stone formation, leading to decreased morbidity and mortality associated with this pathology. Prompt attention to the symptoms produced by urolithiasis is essential to achieve a reduction in incidence; clinicians should be careful not to dismiss symptoms of abdominal pain, haematuria, or dysuria. Another hurdle is neglect and delay in obtaining proper medical advice by the patients and their families; some of this is due to silent stones, but a more important cause is the practice among people, especially from rural background, to consult hakims/quacks for their ailments.

Our study has an advantage over some studies cited above in having a much larger sample size. Since this study was carried out at a dedicated center for kidney disease, the volume of patients is quite high. Not many centers operate in our country providing state-of-the-art care completely free of cost; this means that the catchment area is quite widespread in terms of geography and socioeconomic strata. It is evident from the literature review that not many studies are available regarding the epidemiology of specific diseases in special populations. Our study focuses on a subset within a subset, namely, children ailing with ESRD. While this sort of targeted researches may not be of interest to the casual investigator or practitioner, they are important for those intimately associated with these specialties as they provide important epidemiological data as well as guide important decisions on how to reduce the adverse events patients suffered from during their care.

Unarguably, our study has several limitations as well. The foremost are the facts that this was a retrospective study done in the year 2013; hence, any cases before or after that year were not accounted for. Also, the study was done at a single center. Although the center is located at the city center and would account for most of the cases reported, it still remains our limitation. Our hospital is a government based public institution. Hence, the majority of the children would represent people from a low socioeconomic background.

## 5. Conclusion

Our study highlights a high prevalence of HBV and HCV in paediatric ESRD patients. This unfortunate affliction represents an additional comorbidity for our patients who are already fighting a life-threatening condition. There is a need for carrying out further work to confirm these findings and expand our recommendations, particularly the sensitive issue regarding proper blood screening. NGOs and governmental institutions can play a pivotal role in bringing out the true picture as defined in earlier studies and awareness projects. Further data regarding these infections should be collected on a national level, and appropriate measures should be taken by both the government and private setups to reduce the spread of HBV and HCV as they will have a big effect on the quality of life of patients as well as playing a role in reducing mortality. Hence, focused efforts should be done to prevent the spread of HBV and HCV and thereby reduce the burden of related chronic liver disease in the country and region, as a whole, especially in a region dominated with child population.

## Figures and Tables

**Table 1:** Demographic profile of ESRD children.

	Frequency	Percentage
Age groups (years)		
<1	0	0%
1–9	91	20.5%
10–14	179	40.3%
15–18	174	39.2%
Gender		
Male	262	59.0%
Female	182	41.0%
Province		
Sindh	216	48.6%
Punjab	53	11.9%
KPK	9	2.0%
Baluchistan	24	5.4%
Karachi	137	30.9%
Others	5	1.1%

**Table 2:** Clinical status of ESRD children.

	Frequency	Percentage
Aetiology		
Unknown	294	66.2%
Stone	44	9.9%
Nephrotic	22	5.0%
Glomerulonephritis (nephritic)	22	5.0%
Obstructive uropathy	9	2.0%
SSK (small shrunken kidneys)	28	6.3%
Others (Wilms, BOO, gastroenteritis, etc.)	22	5.0%
VUR (vesicoureteral reflux)	3	.7%
Hepatitis B		
Positive	11	2.5%
Negative	433	97.5%
Hepatitis C		
Positive	13	2.9%
Negative	431	97.1%

**Table 3:** Stratified analysis of clinical status among children with ESRD.

	Age groups (years)					
	1–9		10–14		15–19	
	Frequency	Percentage	Frequency	Percentage	Frequency	Percentage
Aetiology						
Unknown	61	67.0%	116	64.8%	117	67.2%
Stone	10	11.0%	16	8.9%	18	10.3%
Nephrotic	4	4.4%	10	5.6%	8	4.6%
GN (nephritic)	5	5.5%	7	3.9%	10	5.7%
Obstructive uropathy	1	1.1%	6	3.4%	2	1.1%
SSK	5	5.5%	15	8.4%	8	4.6%
Others (Wilms, BOO, gastroenteritis, etc.)	4	4.4%	8	4.5%	10	5.7%
VUR	1	1.1%	1	0.6%	1	0.6%
Hepatitis B						
Positive	1	1.1%	5	2.8%	5	2.9%
Negative	90	98.9%	174	97.2%	169	97.1%
Hepatitis C						
Positive	2	2.2%	3	1.7%	8	4.6%
Negative	89	97.8%	176	98.3%	166	95.4%

## References

[1] Mortazavi F., Maleki M. (2012). Management and outcome of children with end-stage renal disease in northwest Iran. *Indian Journal of Nephrology*.

[2] Groothoff J. W. (2005). Long-term outcomes of children with end-stage renal disease. *Pediatric Nephrology*.

[3] Al-Mugeiren M., Al-Faleh F. Z., Ramia S., Al-Rasheed S., Mahmoud M. A., Al-Nasser M. (1992). Seropositivity to hepatitis C virus (HCV) in Saudi children with chronic renal failure maintained on haemodialysis. *Annals of Tropical Paediatrics*.

[4] Sheth R. D., Peskin M. F., Du X. L. (2014). The duration of hepatitis B vaccine immunity in pediatric dialysis patients. *Pediatric Nephrology*.

[5] Jonas M. M., Zilleruelo G. E., LaRue S. I., Abitbol C., Strauss J., Lu Y. (1992). Hepatitis C infection in a pediatric dialysis population. *Pediatrics*.

[6] Peco-Antić A., Peklar P., Zerjev S. (1993). Viral hepatitis C—a problem in the treatment of children with renal insufficiency on hemodialysis. *Srpski Arhiv Za Celokupno Lekarstvo*.

[7] Greco M., Cristiano K., Leozappa G., Rapicetta M., Rizzoni G. (1993). Hepatitis C infection in children and adolescents on haemodialysis and after renal transplant. *Pediatric Nephrology*.

[8] Callis L. M., Clanxet J., Fortuny G., Caballeria J., Carrasco J. L., Lardinois R. (1985). Hepatitis B virus infection and vaccination in children undergoing hemodialysis. *Acta Paediatrica Scandinavica*.

[10] Rizvi S. A., Manzoor K. (2002). Causes of chronic renal failure in Pakistan: a single large center experience. *Saudi Journal of Kidney Diseases and Transplantation*.

[11] World Health Organization (2012). Hepatitis-C. *Fact Sheet*.

[12] Idrees M., Rafique S., Rehman I.-U. (2009). Hepatitis C virus genotype 3a infection and hepatocellular carcinoma: Pakistan experience. *World Journal of Gastroenterology*.

[13] Jadoon S. A., Jadoon H. A., Nazar H. S. (2014). Treatment of chronic hepatitis-C with standard interferon and ribavirin. *Journal of Ayub Medical College Abbottabad*.

[14] Rodrigo C., Rajapakse S. (2009). Current status of HIV/AIDS in South Asia. *Journal of Global Infectious Diseases*.

[15] Khan H. I. (1996). A study of seroprevalence of hepatitis B and C in mothers and children in Lahore. *Pakistan Pediatric Journal*.

[16] Parker S. P., Khan H. I., Cubitt W. D. (1999). Detection of antibodies to hepatitis C virus in dried blood spot samples from mothers and their offspring in Lahore, Pakistan. *Journal of Clinical Microbiology*.

[17] Hyder S. N., Hussain W., Aslam M., Maqbool S. (2001). Seroprevalence of anti-HCV in asymptomatic children. *Pakistan Journal of Phytopathology*.

[18] Anwar M. I., Rahman M., Hassan M. U., Iqbal M. (2013). Prevalence of active hepatitis C virus infections among general public of Lahore, Pakistan. *Virology Journal*.

[19] Bosan A., Qureshi H., Bile K. M., Ahmad I., Hafiz R. (2010). A review of hepatitis viral infections in Pakistan. *Journal of the Pakistan Medical Association*.

[20] Seck S., Dahaba M., Gueye S., Ka E. (2014). Trends in hepatitis C infection among hemodialysis patients in Senegal: results of a decade of prevention. *Saudi Journal of Kidney Diseases and Transplantation*.

[21] Alashek W. A., McIntyre C. W., Taal M. W. (2012). Hepatitis B and C infection in haemodialysis patients in Libya: prevalence, incidence and risk factors. *BMC Infectious Diseases*.

[22] Huma M. M., Siddiqui D. M., Bashir D. B., Ali D. S., Baloch D. A., Masroor D. M. (2014). Hemodialysis patients profile at Dow University of Health Sciences. *Pakistan Journal of Medical Sciences*.

[23] Hammad A. M., Zaghloul M. H. E. D. (2009). Hepatitis G virus infection in Egyptian children with chronic renal failure (single centre study). *Annals of Clinical Microbiology and Antimicrobials*.

[24] Warady B. A., Chadha V. (2007). Chronic kidney disease in children: the global perspective. *Pediatric Nephrology*.

[25] Harambat J., van Stralen K. J., Kim J. J., Tizard E. J. (2012). Epidemiology of chronic kidney disease in children. *Pediatric Nephrology*.

[26] Hussain M., Lal M., Ali B., Naqvi S. A. A., Rizvi S. A. H. (1998). Urolithiasis in Sindh: a single centre experience with review of 10,000 cases. *Journal of Nephrology, Urology and Transplantation*.

[27] Rizvi S. A. H., Naqvi S. A. A., Hussain Z. (2002). Living-related pediatric renal transplants: a single-center experience from a developing country. *Pediatric Transplantation*.

[28] Rizvi S. A. H., Sultan S., Zafar M. N. (2007). Evaluation of children with urolithiasis. *Indian Journal of Urology*.

[29] Milliner D. S., Murphy M. E. (1993). Urolithiasis in pediatric patients. *Mayo Clinic Proceedings*.

[30] VanDervoort K., Wiesen J., Frank R. (2007). Urolithiasis in pediatric patients: a single center study of incidence, clinical presentation and outcome. *Journal of Urology*.

[31] Goldfarb D. S. (2003). Increasing prevalence of kidney stones in the United States. *Kidney International*.

